# Function of sildenafil on diseases other than urogenital system: An umbrella review

**DOI:** 10.3389/fphar.2023.1033492

**Published:** 2023-02-06

**Authors:** Zeyu Chen, Yin Huang, Dehong Cao, Shi Qiu, Bo Chen, Jin Li, Yige Bao, Qiang Wei, Ping Han, Liangren Liu

**Affiliations:** ^1^ Department of Urology and Institute of Urology and National Clinical Research Center for Geriatrics, West China Hospital, Sichuan University, Chengdu, China; ^2^ West China School of Clinical Medicine, Sichuan University, Chengdu, China

**Keywords:** sildenafil, phosphodiesterase-5 inhibitor, therapeutic function, vascular dysfunction, umbrella review

## Abstract

**Background:** To investigate the function of sildenafil on diseases other than urogenital system, an umbrella review was conducted.

**Methods:** Meta-analysis and systematic reviews on this topic were comprehensively evaluated in this umbrella review. Quality of evidence was evaluated through AMSTAR and the Grading of Recommendations, Assessment, Development and Evaluation system to generate a reliable and valid conclusion.

**Results:** 77 out of 1164 meta-analysis were enrolled. 33 significant outcomes and 41 non-significant outcomes were extracted from all eligible articles. We found sildenafil did significant help in reducing arterial systolic pressure, mean pulmonary arterial pressure, pulmonary arterial pressure, systolic pulmonary arterial pressure in patients with pulmonary and cardiovascular diseases. Besides, sildenafil also improved exercise capacity or performance in patients with pulmonary and cardiovascular diseases. Other than these patients, this drug contributed great help in pregnant women with fetal growth restriction and preeclampsia by increasing the weight of newborns and lowering uterine and umbilical pulsatility indices. Additionally, it was reported that utilization of sildenafil has brought increased risk of melanoma.

**Conclusion:** We can conclude from our study that sildenafil played an important role in many fields, especially in vascular protection. This finding provides a strong evidence for further expansion of sildenafil utilization in other diseases.

## 1 Background

Sildenafil, marketed as Viagra, is a highly selective phosphodiesterase-5 (PDE-5) inhibitor. It was first developed by Pfizer to augment the vasodilation induced by nitric oxide and relieve angina in ischemic heart disease. However, interestingly, this drug was first approved by the FDA preliminarily for use as a medication intervention in 1998 in the treatment of male erectile dysfunction (Boolell et al., 1996; Langtry and Markham, 1999). Most formulations on the market consist of film‐coated tablets, it is relatively rapidly absorbed after taken orally and reaching the maximum plasma concentration between 30 min and 2 h (median Tmax = 0.8–1 h). Besides, the bioavailability of sildenafil is approximately 40% because it was metabolized by cytochrome P450 enzymes in the liver (Hong et al., 2017). Sildenafil shared multiple structural and functional features with vardenafil, tadalafil and avanafil. Despite these common features shared by PDE5-inhibitors, differences existed between these agents and led to differentiated selectivity, potency, indication and duration etc (Ahmed et al., 2021). Sildenafil was reported to have low activity against PDE6 and very low activity against PDE1, vardenafil had similar selectivity as sildenafil because of similar structural and chemical features. Tadalafil could act with PDE family except for PDE11 and PDE6. Avanafil was the only second generation and most selective PDE5-inhibitor among these four drugs (Andersson, 2018). Previous studies have reported similar function as vasorelaxants by relaxing and dilating smooth muscle cells. Phosphodiesterase-5 is expressed widely in various organs, such as the corpus cavernosum, blood vessels, uterus, liver, and kidney (Lin, 2004; Lin et al., 2006) and distributed approximately equally high in lung and penile corpus cavernosum, function of PDE5-inhibitors in pulmonary vascular might be as good as in erectile dysfunction (Corbin et al., 2005). According to existing evidence, all four drugs were approved for application in erectile dysfunction, sildenafil and tadalafil was approved for therapy of PH, tadalafil was the only one could be used in lower urinary tract symptoms caused by benign prostatic hyperplasia (Ahmed et al., 2021).

As the first and most representative PDE5-inhibitor, sildenafil presented good vasorelaxation function through the regulation of cGMP-NO pathway induced by inhibition of PDE5 (Langtry and Markham, 1999). As described previously, the wide distribution of PDE5 indicated expansion of the utilization of sildenafil. Investigations and clinical trials on the use of sildenafil in other fields have been conducted widely in recent years. Its function in the treatment of pulmonary arterial hypertension is the most well-studied and has become another indication of this drug in recent years. Studies in these fields are still ongoing, and most of them are randomized controlled trials based on limited cohort sizes. Besides, investigations on function of sildenafil on myocardial diseases, diseases on endocrine system, fetal growth restriction, cancers *etc.* were also conducted. Some meta-analyses reported pooled results of these RCTs in a certain field. However, similar studies on other PDE5-inhibitors are still limited. To the best of knowledge, no attempt has been made to systematically summarize the comprehensive function of sildenafil in diseases other than the urogenital system. Based on this background, we are aiming to conduct a comprehensive evaluation of the function of sildenafil reported by systematic reviews and meta-analyses.

To provide a general evaluation of the quality of evidence, possible biases and validity of the function of sildenafil, we performed this umbrella review of the evidence according to existing systematic reviews and meta-analyses.

## 2 Methods

### 2.1 Umbrella review methods

Meta-analyses and systematic reviews on the function of sildenafil in multiple diseases were systematically searched, organized and evaluated in our research (Aromataris et al., 2015; Papatheodorou, 2019). Systematic reviews without meta-analyses were excluded from our study (Poole et al., 2017). The utilization of sildenafil was under the prescription directed by doctors (investigators).

### 2.2 Literature search

We searched Medline, Embase, the Cochrane Database of Systematic Reviews and Web of Science from inception through April 2022 for systematic reviews and meta-analyses of observational or interventional studies. The following search strategy was used: (sildenafil OR Viagra) AND (systematic review OR meta-analysis). The SIGN guidance for systematic reviews and meta-analyses was used for the literature search (Li et al., 2020). Two investigators (ZYC and YH) screened the titles and abstracts independently and selected eligible articles through full text review. Any discrepancies in the selection of articles between the two researchers were resolved by a third investigator (LRL). The references cited in all eligible articles were also manually searched.

### 2.3 Eligibility criteria

Systematic reviews with meta-analyses of observational (cohort and case‒control) and interventional studies (randomized and non-randomized controlled trials) assessing the function of sildenafil on diseases were included. Articles were included if the exposure was sildenafil regardless of the race, gender, country or region of participants. If an article reported two or more outcomes, we extracted the data of each outcome separately. If a single parameter was investigated by two or more studies, we selected the one with a larger number of participants. In addition, meta-analyses of total PDE5is or including other drugs were excluded unless we could obtain data on sildenafil usage separately through the subgroup analysis. Studies focused on the effect of sildenafil on diseases of the urogenital system were also excluded. We also excluded studies published in languages other than English, animal and laboratory studies.

### 2.4 Data extraction

CZY and HY independently extracted the following data from eligible studies: 1) name of the first author, 2) journal, 3) year of publication, 4) outcome, 5) number of included studies, 6) number of participants in each study, 7) study design (case‒control, cohort, randomized controlled trial (RCT) and non-randomized controlled trial), 8) the estimated summary effect (RR, relative risk; OR, odds ratio; SMD; WMD; Hedge’s G; SD; MD; IV) and corresponding 95% confidence intervals (CIs). In addition, we extracted the type of effect model (fixed or random), I^2^ statistic, Cochran’s Q test *p*-value and publication bias by Egger’s test if available. Any difference was resolved by the third investigator (LRL).

### 2.5 Assessment of methodological quality of included studies and quality of evidence

We evaluated the methodological quality of the included articles through AMSTAR by eleven items, which is reliable and valid in assessing the quality of systematic reviews and meta-analyses (Shea et al., 2007). The Grading of Recommendations, Assessment, Development and Evaluation (GRADE) was used to assess the strength of evidence for each outcome presented in the umbrella review and to classify evidence into “high”, “moderate”, “low” and “very low” quality to make recommendations (Guyatt et al., 2011).

### 2.6 Data analysis

We extracted the exposure and outcome data and estimated the summary effect with a 95% confidential interval (CI) reported in each meta-analysis if available (Poole et al., 2017; Li et al., 2021). If an article included meta-analyses of both cohort and case‒control studies and analysis was only performed separately without overall outcome, we extracted the data by study design. We performed the I^2^ statistic and Cochran’s Q test as an estimate of the heterogeneity between studies. The estimate of publication bias in each study was calculated by Egger’s regression test (Egger et al., 1997). If available, dose–response relations in meta-analyses were also presented. A *p*-value <0.10 was regarded as significant for Egger’s test and heterogeneity. In addition, a *p*-value <0.05 was regarded as significant for other tests.

## 3 Results

### 3.1 Characteristics of the included meta-analyses

**FIGURE 1:**
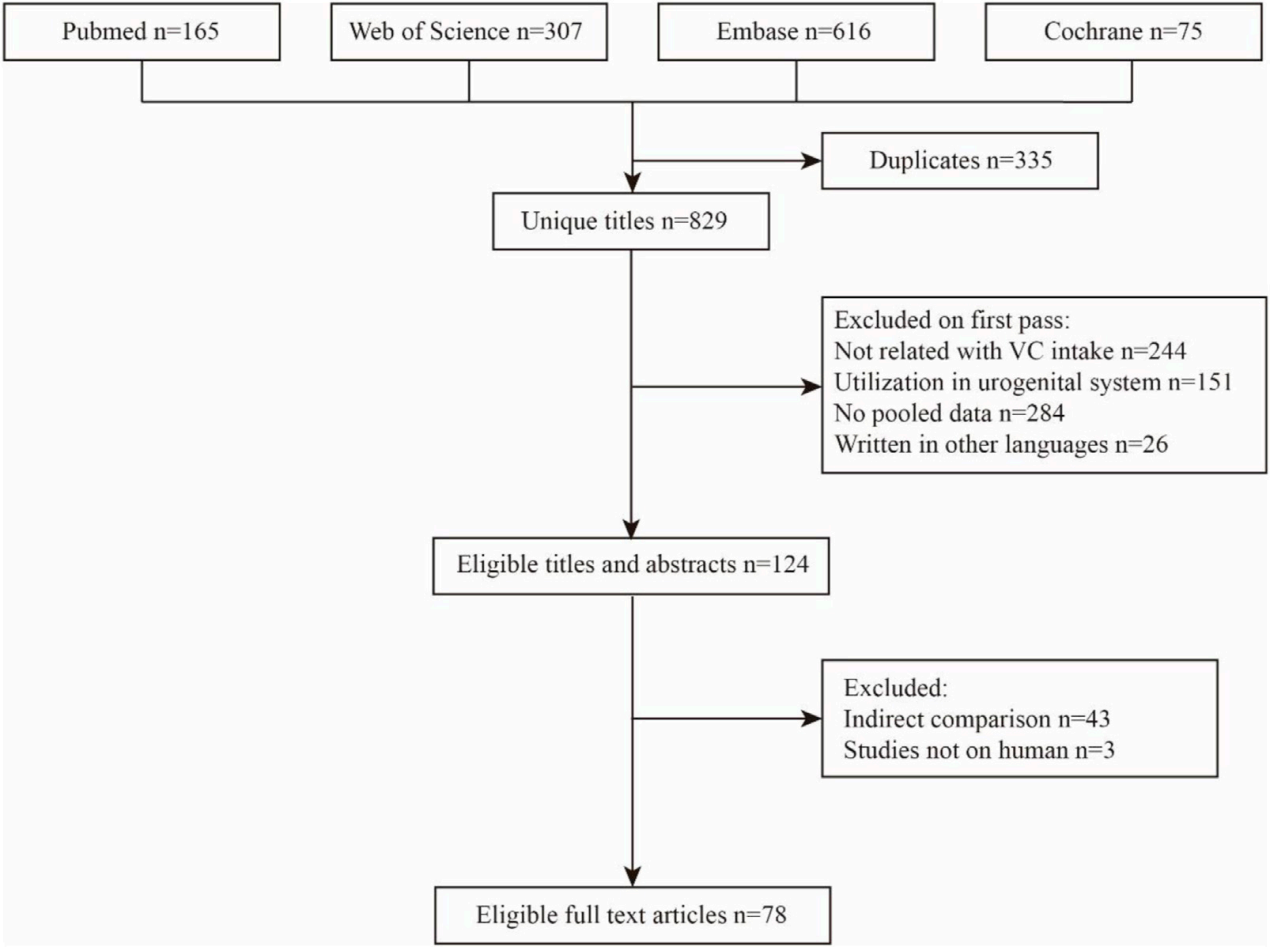
Process of the systematic search and selection.

The detailed process of the literature search and selection is presented as a flow chart in Figure 1. We obtained 1,164 articles from the most used databases and finally identified 77 meta-analyses according to our inclusion and exclusion criteria. Thirty-three significant sildenafil therapeutic outcomes and 41 non-significant outcomes of diverse diseases were extracted from all eligible studies. Data of significant outcomes are tabulated in Table 1 and information of non-significant outcomes are in Supplementary Table S1.

**TABLE 1:** Significant effect of sildenafil on multiple diseases.

Outcome	Ref. no.	Study population	No. of case/control	MA metrics	Estimates	95%CI	No. of studies	RCT	Observational studies	Effect model	I^2^; Q test *p*-value	Egger test *p*-value
Cardio-respiratory system												
** **6-min walk distance (meters)	13	COPD with PH	291/288	WMD	29.64	13.78, 45.5	9	9	—	Random	90%; <0.00001	—
** **Pulmonary arterial systolic pressure (mmHg)	13	COPD with PH	244/247	WMD	−7.86	−11.26, −4.46	7	7	—	Random	71%; 0.002	—
** **Pulmonary arterial systolic pressure (mmHg)	14	HF with reserved ejection fraction	101/100	SMD	−9.02	−14.51, −3.54	5	5	—	Random	94%; <0.00001	0.001
** **Pulmonary arterial systolic pressure (mmHg)	14	HF	135/133	SMD	−7.51	−12.70, −2.32	6	6	—	Random	94%; <0.00001	0.005
** **Pulmonary arterial systolic pressure (mmHg)	15	PH with left heart disease	133/127	WMD	−7.80	−12.28, −3.32	4	4	—	Random	97%; <0.00001	0.00006
** **Pulmonary arterial systolic pressure (mmHg)	16	PH	29/29	WMD	−11.14	−17.56, −4.72	2	2	—	Random	73%; 0.06	0.00067
** **Mean pulmonary arterial pressure 24 h after treatment (mmHg)	17	Neonates with persist PH	32/25	SMD	−1.87	−2.50, −1.23	2	2	—	Fixed	29.7%; 0.233	
Mean pulmonary arterial pressure (mmHg)	18	pediatric PH	337	RR	−0.31	−0.54, −0.08	7	7	—	Random	84.6%; 0.000	
** **Mean pulmonary arterial pressure (mmHg)	15	PH with left heart disease	32/32	WMD	−3	−4.66, −1.34	2	2	—	Fixed	0%, 1.0	0.0004
** **Mean pulmonary arterial pressure (mmHg)	19	PH secondary to chronic systolic heart failure	48/48	WMD	−5.71	−11.37, −0.06	3	3	—	Random	86%; 0.0006	0.048
** **Pulmonary arterial pressure during rest (mmHg)	20	hypoxia	41/42	Hedge’s G	−1.52	−2.23, −0.82	7	7	—	Random	76.%; <0.05	
** **Pulmonary arterial pressure during exercise (mmHg)	20	hypoxia	28/29	Hedge’s G	−1.19	−2.13, −0.26	4	4	—	Random	81.5%; <0.05	
pre- and intra-operative systolic pulmonary arterial pressure (mmHg)	21	PH undergoing cardiac surgery	76/77	WMD	−11.19	−20.23, −2.15	3	3	—	Random	62%; 0.07	0.02
** **Pre- and post-operative systolic pulmonary arterial pressure (mmHg)	21	PH undergoing cardiac surgery	50/50	WMD	−13.67	−19.56, −7.78	2	2	—	Random	0%; 0.54	<0.00001
** **Partial pressure of O_2_ 24 h (mmHg)	17	neonates	33/25	SMD	0.86	0.31, 1.41	2	2	—	Fixed	0.0%; 0.563	
** **Oxygenation index 24 h (mmHg)	17	neonates	39/30	SD	−1.51	−2.07, −0.95	3	3	—	Random	69.2%; 0.039	
** **Pulmonary vascular resistance (Wood unit m^2^)	19	PH secondary to chronic systolic heart failure	54/54	WMD	−81.50	−104.29, −12.79	3	3	—	Random	0%; 0.43	<0.00001
** **Pulmonary vascular resistance (Wood unit m^2^)	15	PH with left heart disease	32/32	WMD	−60.0	−105.51, −14.49	2	2	—	Fixed	0%, 1.0	0.01
** **Oxygenation index for neonates (6–7 h) (mmHg)	22	Infants with PH	40/37	WMD	−20.07	−26.12, −14.02	2	2	—	Fixed	0%; 0.47	<0.00001
Mean airway pressure (24 h) (cmH_2_O)	22	Infants with PH	32/25	WMD	−6.64	−8.49, −4.8	2	2	—	Fixed	0%; 0.34	<0.00001
** **Post-operative inotrope requirement	21	PH undergoing cardiac surgery	46/47	RR	0.38	0.2, 0.74	2	2	—	Random	0%, 0.71	0.004
** **Hospitalization	19	PH secondary to chronic systolic heart failure	79/78	RR	0.29	0.11, 0.77	4	4	—	Random	0%; 0.99	0.01
** **Left ventricular ejection fraction (%)	19	PH secondary to chronic systolic heart failure	61/60	WMD	3.95	1.45, 6.44	3	3	—	Random	67%; 0.05	0.002
** **Left ventricular ejection fraction (%)	14	HF with reserved ejection fraction	46/45	SMD	5.89	4.01, 7.78	2	2	—	Fixed	0%; 0.92	<0.00001
** **Left ventricular ejection fraction (%)	14	HF	80/78	SMD	5.43	3.66, 7.20	3	3	—	Fixed	0%; 0.37	<0.00001
** **Oxygen consumption at peak exercise (Peak VO_2_) (L/min)	19	PH secondary to chronic systolic heart failure	105/101	WMD	3.2	2.72, 3.67	6	6	—	Random	0%; 0.76	<0.00001
** **Peak VO_2_ (L/min)	14	HF	178/178	SMD	2.14	0.08, 4.21	6	6	—	Random	83%; <0.0001	0.04
** **Ventilation to CO_2_ production slope (VE/VCO_2_ slope)	19	PH secondary to chronic systolic heart failure	88/84	WMD	−5.89	−7.13; −4.65	5	5	—	Random	31%; 0.21	<0.00001
** **VE/CO_2_ slope	14	HF	73/69	SMD	−7.06	−8.93, −5.19	4	4	—	Fixed	20%; 0.29	<0.00001
** **VO_2_ at anaerobic threshold (%)	14	HF	62/61	SMD	3.47	1.68, 5.27	3	3	—	Fixed	0%; 0.67	0.0002
** **Flow-mediated dilation (FMD) values (% of basal)	28	type 2 diabetes mellitus	62/56	WMD	2.21	0.41, 4.01	4	4	—	Random	98%; <0.00001	0.02
SPO_2_ (%)	20	hypoxia	112/113	Hedge’s G	0.47	0.22–0.73	14	14	—	Random	43.7%	
** **Breathless	19	PH secondary to chronic systolic heart failure	62/61	WMD	7.72	5.85, 9.59	3	3	—	Random	0%; 0.73	<0.00001
** **Fatigue	19	PH secondary to chronic systolic heart failure	62/61	WMD	2.28	0.01, 4.55	3	3	—	Random	0%; 0.48	0.049
** **Emotional function	19	PH secondary to chronic systolic heart failure	62/61	WMD	5.92	3.37, 8.47	3	3	—	Random	28%; 0.25	<0.00001
** **Mortality	18	pediatric PH	237/264	RR	0.25	0.12, 0.51	11	11	—	Fixed	0%; 0.67	
** **Clinical worsening	24	PH	351/213	OR	0.34	0.18, 0.64	3	3	—	Random	0%; 0.49	0.0009
** **Length of ICU stay (hours)	25	children with PH secondary to congenital heart disease	59/61	MD	−21.84	−29.15, −14.53	3	3	—	Fixed	0%; 0.69	<0.00001
** **Cardiac output during exercise (L/min)	20	hypoxia	63/63	Hedge’s G	0.3	0.01–0.59	9	9	—	Random	29.4%	
** **Cardiac output during rest (L/min)	20	hypoxia	47/48	Hedge’s G	0.55	0.06, 1.04	4	4	—	Random	65.3%	
** **Exercise capacity	23	COPD with PH	111/113	MD	−9.55	−11.42, −7.68	4	4	—	Random	74%; 0.009	
** **Performance	20	hypoxia	77/77	Hedge’s G	0.47	0.01–0.94	8	8	—	Random	75.4%	
Pregnancy												
Fetal weight g)	26	pregnant women	255/253	WMD	222.58	27.75, 417.41	5	5	—	Random	96%; <0.00001	0.03
Uterine artery pulsatility indices (%)	27	pregnancies with fetal growth restriction	245/253	WMD	−0.39	−0.48, −0.30	3	3	—	Random	0.0%; 0.54	
Umbilical artery pulsatility indices (%)	27	pregnancies with fetal growth restriction	245/253	WMD	−0.08	−0.17, 0.00	6	6	—	Random	74.6%; 0.001	
Cancer												
Melanoma risk	29	75631/825518	RR	1.26	1.07, 1.5	6	—	Random	79%; 0.0002	0.007		

Abbreviations: COPD, chronic obstructive pulmonary disease; PH, pulmonary arterial hypertension; HF, heart failure; WMD, weighted mean difference; SMD, standard mean difference; RR, risk ratio, Hedge’s G Hedge’s Glass’s estimator, SD, standard difference; OR, odds ratio; MD, mean difference.

### 3.2 Effect of sildenafil on cardiovascular and pulmonary diseases

Sildenafil brought significant benefit to patients with cardiovascular dysfunction. This therapeutic effect is often evaluated through hemodynamic parameters. Decreased pulmonary arterial systolic pressure was observed in chronic obstructive pulmonary disease (COPD) with pulmonary hypertension (PH) (WMD: −7.86; 95% CI: −11.26, 4.46) (Hao et al., 2020), heart failure (HF) with reduced ejection fraction (SMD: 9.02; 95% CI: −14.51, 3.54) (Zhuang et al., 2014), HF (SMD: −7.51; 95% CI: −12.70, 2.32) (Zhuang et al., 2014), PH with left heart disease (WMD: −7.80; 95% CI: −12.28, −3.32) (Jiang et al., 2015) and PH (WMD: −11.14; 95% CI: −17.56, −4.72) (Kanthapillai et al., 2004) patients after sildenafil intervention compared with placebo. Sildenafil helped to lower the mean pulmonary arterial pressure of patients with neonatal PH (SMD: −1.87; 95% CI: −2.50, −1.23) (He et al., 2021), pediatric PH (RR: 0.31; 95% CI: −0.54, −0.08) (Zhang et al., 2020), PH with left heart disease (WMD: 3; 95% CI: −4.66, −1.34) (Jiang et al., 2015) and PH secondary to chronic systolic heart failure (WMD: −5.71; 95% CI: −11.37, −0.06) (Wu et al., 2014). Another meta-analysis investigated the effect of sildenafil on hypoxia patients, and pulmonary arterial pressure decreased to 1.52 (Hedge’s G: −1.52, 95% CI −2.23, −0.82) and 1.19 (Hedge’s G: 1.19, 95% CI −2.13, −0.26) during rest and exercise, respectively (Carter et al., 2019). For PH patients undergoing cardiac surgery, this drug could lower the intra- (WMD: 11.19; 95% CI: −20.23, −2.15) and post- (WMD: −13.67; 95% CI: −19.56, −7.78) operative systolic pulmonary arterial pressure compared with preoperative (Villanueva et al., 2019a). Additionally, preoperative sildenafil intervention could greatly reduce the requirement of inotrope after surgery (RR: 0.38, 95% CI: 0.2, 074). Sildenafil could reduce pulmonary vascular resistance in PH patients secondary to chronic high systolic pressure (WMD: −81.50; 95% CI: −104.29, −12.79) (Wu et al., 2014) and PH with left heart diseases (WMD: −60.0; 95% CI: −105.51, −14.49) (Jiang et al., 2015). Sildenafil also greatly increased left ventricular ejection fraction in patients with PH secondary to chronic systolic heart failure and heart failure. This management could also help with patients’ respiratory function. Two meta-analyses reported a significantly lowered oxygenation index for neonates with PH with their pooled data from several RCTs (Kelly et al., 2017; He et al., 2021); one of these two studies also proposed an increase in the partial pressure of O_2_ (He et al., 2021). Type II diabetes is a chronic disease and can affect many systems due to hyperglycemia, including the cardiovascular system. To evaluate the damage to cardiovascular function caused by hyperglycemia, we always use several hemodynamic parameters. The utility of sildenafil in patients with type 2 diabetes mellitus was related to the recovery of vascular epithelial function (flow-mediated dilation increased by 2.21 (WMD: 2.21, 95% CI: 0.41, 4.01)) (He et al., 2010).

Regarding the estimation of people’s exercise capacity, an increased performance of 6-min walk distance was observed in COPD patients with PH who received sildenafil (WMD: 29.64; 95% CI: 13.78, 45.50) (Hao et al., 2020) compared with the placebo group. Considering demographic estimations, sildenafil increased peak oxygen uptake in patients with PH secondary to chronic systolic heart failure (WMD: 3.2, 95% CI: 2.72, 3.67) and heart failure (SMD: 2.14, 95% CI: 0.08, 4.21) compared with placebo (Wu et al., 2014; Zhuang et al., 2014), and oxygen consumption at the anaerobic threshold of heart failure patients increased as well (SMD: 3.47, 95% CI: 1.68, 5.27) (Zhuang et al., 2014). The VE/VCO_2_ slope is also a crucial method to evaluate people’s exercise capacity; patients in the sildenafil group showed a significantly lower VE/VCO_2_ slope in patients with PH secondary to chronic systolic heart failure (WMD: −5.89, 95% CI: −7.13, −4.65) (Wu et al., 2014) and heart failure (SMD: −7.06, 95% CI: −8.93, −5.19) (Zhuang et al., 2014). Sildenafil intervention also enhanced oxygen saturation in patients with hypoxia (Hedge’s G: 0.47, 95% CI 0.22, 0.73) (Carter et al., 2019) and led to elevated cardiac output during rest or exercise in the same cohort, which may explain the improvement in their daily performance.

Other than these objective parameters, sildenafil intake could ease cardio-pulmonary disease-related symptoms: breathlessness, fatigue, emotional function and exercise capacity (Wu et al., 2014; Chen et al., 2015). This drug could also lower the possibility of clinical worsening for PH patients (He et al., 2010), length of ICU stays for children with PH secondary to congenital heart disease (Jiang et al., 2018a) and mortality of children with PH (Zhang et al., 2020).

### 3.3 Effect of sildenafil on pregnancies

Sildenafil also contributed great help for pregnant women with fetal growth restriction or preeclampsia. A 222.58 g (WMD: 222.58, 95% CI: 27.75, 417.41) increase in the weight of newborns was observed in pregnant women who received sildenafil compared with placebo (Ferreira et al., 2019). For pregnant women with fetal growth restrictions, sildenafil could lower uterine pulsatility indices by 0.39 (WMD: −0.39, 95% CI: −0.48, 0.30), and umbilical pulsatility indices were reduced by 0.08 (WMD: −0.08, 95% CI: −0.17, 0) (Hessami et al., 2021).

### 3.4 Effect of sildenafil on melanoma

Other than the functions mentioned above, limited evidence indicated that a melanoma-promoting function in participants using sildenafil was reported by Han et al. in their pooled data from six large-scale observational studies; 75631 participants using sildenafil had a 0.26-fold increased risk of melanoma compared with 825518 with placebo (RR: 1.26, 95% CI: 1.07, 1.5) (Han et al., 2018). However, no pooled evidence was found to explain the association between sildenafil utilization and other malignancies.

### 3.5 Non-significant effect of sildenafil on multiple diseases

As shown in Supplementary Table S1, sildenafil did no effect on the 6-min walk distance and Borg score at rest in patients with fibrotic interstitial lung disease (Bajwah et al., 2013) and SF-36 survey scale as well as Borg-dyspnea index results in COPD patients with PH(17). It was also reported that no significant change in mean pulmonary arterial blood pressure and alveolar‐arterial oxygen difference in neonates and infants with PH treated with sildenafil (Kelly et al., 2017; He et al., 2021). In children with or without PH secondary to congenital heart disease, sildenafil did little help in mechanical ventilation time, length of ICU, incidence of pulmonary hypertensive crisis, length of hospital stay, mortality before discharge and time on the length of hospitalization (Jiang et al., 2018b; Zhang et al., 2020). This drug did not induce significant improvement in some hemodynamic parameters (pulmonary capillary wedge pressure, pre- and intra-operative mean pulmonary arterial pressure, pre- and intra-operative pulmonary vascular resistance, pre- and intra-operative systemic vascular resistance, systolic and diastolic blood pressure, heart rate and systemic vascular resistance) in patients with HF, PH due to left heart disease, PH undergoing cardiac surgery and PH secondary to chronic systolic heart failure (Wu et al., 2014; Jiang et al., 2015; Villanueva et al., 2019b).

In pregnant women, sildenafil treatment did not achieve significant improvement in gestational age at birth, umbilical artery pulsatility index, indication of delivery due to fetal distress, indication of labor due to maternal laboratory test abnormality, indication of delivery due to imminent eclampsia, neonatal mortality and middle cerebral artery pulsatility index (Ferreira et al., 2019). Besides, it is no efficient in controlling parameters other than hemodynamic system (HbA1c, endothelin 1 serum level, high sensitivity C-reactive protein plasma level and interleukin six serum level) in type II diabetes patients (Santi et al., 2015; Poolsup et al., 2016). Lastly, according to the pooled results of a meta-analysis, sildenafil did not lower the risk of colorectal cancer based on results of amount of population based studies (Bhagavathula et al., 2021).

### 3.6 Heterogeneity and publication bias of included studies

Thirteen out of all 17 included meta-analyses showed Q-test *p* < 0.10. Ten meta-analyses were found to have low levels of heterogeneity (I^2^ < 25%). Publication bias was detected in 12 articles of all, whereas this was not detected in five studies.

### 3.7 AMSTAR and GRADE evaluation of included studies

AMSTAR scores were estimated in our umbrella review, ranging from 5 to 10 points (median 8, interquartile range 7–8), which is relatively good. Supplementary Table S2 shows the detailed AMSTAR scores for each outcome. The quality of 19 pieces of evidence of all included clinical outcomes was identified as “moderate” or “high” in our research. Detailed information on GRADE scores for each outcome is presented in Supplementary Table S3.

## 4 Discussion

Sildenafil has been the predominant agent in the treatment of erectile dysfunction for decades, and its utilization in the urogenital system has also been well studied. In this review, we comprehensively summarized the usage and effect of sildenafil in the management of diseases other than the urogenital system. Sildenafil, as previously described, acts by inhibiting the function of phosphodiesterase-5, which is widely expressed in blood vessels and induces muscle relaxation. This process is mainly regulated by the NO-cGMP pathway. The existence of cGMP was a part of the NO signaling pathway and could activate cGMP-dependent protein kinase. This protein kinase reduced the intracellular calcium concentration and inhibited the actin-myosin cross-bridge cycle, thus inducing smooth muscle relaxation (Kressler et al., 2011). PDE-5 participates in the conversion of cGMP into GMP. Sildenafil could inhibit the catabolizing of cGMP, thus preserving more cGMP and activating more cGMP-dependent protein kinase. In addition, it was also reported to decrease interleukin-6 (IL-6), C-reactive protein (CRP), fibrinogen, and tumor necrosis factor-a (TNF-a) in the blood (Vlachopoulos et al., 2015).

As summarized by our research, sildenafil presented good function in pulmonary-cardiovascular disease control. PH is a disease caused by increased resistance of the pulmonary artery, and the presence of this resistance is mainly due to dysfunction of blood vessel endothelial cells. Endothelial dysfunction is mostly associated with decreased vasodilator production/bioactivity, increased production of vasoconstrictors, vascular smooth muscle hypertrophy and blood vessel remodeling. Elevated pulmonary arterial pressure may cause heart failure (usually starting from the right ventricle) (Schermuly et al., 2011; Han et al., 2018). In addition to relaxation of blood vessel smooth muscle, sildenafil could suppress inflammation of vessels and inhibit artery remodeling (Bogdan et al., 2012). Researchers also proposed the cardiac protection function of sildenafil. Westermann et al. used sildenafil in an angiotensin-II-induced heart failure mouse model and found significantly improved systolic and diastolic function in these mice and associated with decreased cardiac hypertrophy and cardiomyoapoptosis (Westermann et al., 2012). Amelioration of early molecular alterations of the left ventricle (extracellular regulated protein kinases and calcineurin pathway) was also observed. In addition, another study found that sildenafil could alter the metabolism of the myocardium in piglets, and aerobic metabolism was increased by using this drug (Zhang et al., 2014).

The therapeutic effect of sildenafil on PH patients was interpreted by its cardiovascular protective function. Moreover, it also brought benefits to patients with respiratory inflammatory diseases. As described above, sildenafil could inhibit the expression of multiple inflammatory markers; similarly, it could also reduce the production of many inflammatory mediators and regulate several intracellular molecules, such as MAPK, NF-kβ and extracellular regulated protein kinase. Researchers have conducted many experiments dependent on animals to examine the underlying mechanism of its lung disease resistance function. De Visser and his colleagues found alleviated bronchopulmonary dysplasia in rat pups exposed to hypoxia; in this model, increased cGMP and alveolarization, improved angiogenesis, decreased fibrin deposition and erupted inflammation were also found in these rats (de Visser et al., 2009). In another study on bronchopulmonary dysplasia, Park et al. constructed a similar neonatal rat model and found upregulated expression of HIF-1α and VEGF in the lungs of these rats. They tried to verify this finding in human-origin cells (small-airway epithelial cells) and found that sildenafil-induced cGMP accumulation activated HIF-mediated hypoxic signaling by stimulating the PI3K-Akt-mTOR pathway. In another study conducted by Wang et al., they found an increase in cGMP in rat lung tissue, decreased lung NO metabolites, and less leukocyte and cytokine release in bronchoalveolar lavage fluid in an acrolein-induced airway inflammation rat model with units pretreated with sildenafil (Wang et al., 2009). Yildirim and others proposed that sildenafil citrate administration in a bleomycin-induced lung fibrosis rat model could release lung fibrosis by inhibiting lipid peroxidation, cytokine production and/or release and neutrophil accumulation (Yildirim et al., 2010). These effects were all possibly related to the NO/cGMP pathway.

During pregnancy, the exchange of nutrients and substrates over the fetal-maternal interface was guaranteed. Blood perfusion of uteroplacental and umbilical placental tissues was a crucial factor for this activity (Lyall, 2003). Reduction of uteroplacental blood flow could be widely seen in a large proportion of fetal growth restriction and preeclampsia. This hypoperfusion of the placenta increased the production of response oxygen species and decreased NO synthesis as well as increased PDE-5 activation, which could lead to reduced vasodilation (Neilson and Alfirevic, 1996). This similar underlying mechanism indicated similar effectiveness of sildenafil in the treatment of pulmonary/cardiovascular diseases and pregnancies with preeclampsia/fetal growth restriction. Many studies (clinical trials and meta-analyses based on them) have tried to demonstrate the effect of sildenafil on pregnancies. We first comprehensively summarized these findings and proposed that sildenafil increased the weight of newborns and ease the restriction of uteroplacental blood perfusion. This effect was also proven by an animal study, and improved umbilical artery circulation was observed in pregnant mice treated with sildenafil; furthermore, increased fetal weight was also seen in these mice (Stanley et al., 2015).

Type 2 diabetes, as a highly prevalent chronic health concern worldwide, is always associated with vascular damage at an advanced stage. This damage is commonly thought to be caused by hyperglycemia and the development of insulin resistance, thus promoting atherogenesis through cell proliferation at the vascular wall and inducing endothelial damage (Matthaei et al., 2000). This change may result in cardiovascular accidents (Haffner et al., 1998). Previous studies have demonstrated that the mechanism of this vascular endothelial damage was similar to other cardiovascular damages through the NO-cGMP pathway (Haffner et al., 1998). Antioxidant and glycation pathways were activated in diabetic patients, and abnormal production of reactive oxygen species and reduction of NO led to vascular endothelial dysfunction (Hakim and GoldsteIn, 1996; Taylor, 2001; Wen et al., 2002; Hellsten et al., 2012). Due to the *in vivo* presence of this molecular process, impaired hemodynamic parameters (such as FMD) could be observed in these patients. As described above, sildenafil inhibited cardiovascular damage by enhancing the NO-cGMP signaling cascade; this change mainly resulted in the augmentation of vasodilation. The vascular protection ability of sildenafil could be explained by its antioxidant function, such as inhibition of NADPH oxidase activity and reduction in superoxide formation (Milani et al., 2005; Schäfer et al., 2008). This was proven by an animal test; in their test, diabetic rats using sildenafil showed significantly increased total antioxidant capacity over those without sildenafil intervention (Milani et al., 2005).

Our research showed that the utilization of sildenafil is correlated with an increased incidence of melanoma in males. However, existing evidence has proposed a controversial function of sildenafil in the process of melanoma. The potential mechanism may be related to its regulatory function on cGMP (PDE5A-cGMP) (Arozarena et al., 2011). It was reported previously that oncogenic BRAF upregulates the expression of many genes, but a much smaller number of genes were downregulated (PDE5A-packer). CGMP-specific phosphodiesterase (PDE5A) was one of these downregulated genes. This downregulation led to increased cGMP and Ca^2+^ and the induction of invasion through increased cell contractility and inhibited the effect of the RAS/RAF/ERK/MEK pathway (Packer et al., 2009; Flaherty and McArthur, 2010). Similar to other PDE5is, PDE5A is also the predominant target gene of sildenafil. The pharmacologic function of sildenafil mimics this inhibition in melanocytes and thus contributes to the initiation and progression of melanoma.

According to the result of previous studies, the most common seen adverse effect of sildenafil is headache, and other common adverse effects included: dizziness, abnormal vision, flushing, nasal congestion, nausea, dyspepsia (Pfizer Inc., 2007). These findings suggested usage of sildenafil is relatively safe under appropriate prescription. Data of its safety in women of childbearing potential is still limited (Pfizer Inc., 2007), and no clinically significant side effect was observed in a meta-analysis by He et al. for treating persistent pulmonary hypertension in neonates (He et al., 2021). Notably, few drugs were reported to have significant adverse interactions with sildenafil, the most commonly reported are nitrates, cytochrome P450 3A4 (CYP3A4) inhibitors and alpha-blockers (Hong et al., 2017). All PDE5is could enhance the vasodilatory and hypotensive function of nitrates and NO donors. As a result, it is recommended to wait for at least 24–48 h prior to taking nitrates for those had taken PDE5is according to the American College of Cardiology, furthermore, a close hemodynamic monitoring should be performed (Abrams, 2004). According to the pharmacokinetic theory of sildenafil, it is primarily metabolized *via* CYP3A4, a potential drug interaction is with strong CYP3A4 inhibitors, which could cause elevated and prolonged serum concentrations of PDE5is and may resulted in hemodynamic disturbance (Mehrotra et al., 2007). Due to the hypotensive effect of sildenafil, the concomitant utilization of alpha-blockers may lead to orthostatic hypotension. Patients using alpha-blockers may start sildenafil treatment at the lowest dose (Kloner, 2005).

We noticed some outcomes existed not only in the significant reports, but in the non-significant studies as well. This repeated appearance of a single outcome could be explained by the diversion of their study population. In our study, if an outcome on the same study population appeared in more than one article, we would select the one with the largest cohort size or the latest time (always regarded as the most representative) (Poole et al., 2017; Papatheodorou, 2019). This would guarantee the quality of our evidence and provide credible support to our results. And some non-significant outcomes did not reach statistical significance might because of the limited cohort size of RCT.

Our umbrella review is the first comprehensive overview of the published literature and current evidence on the function of sildenafil in multiple diseases. We conducted this umbrella review through strict and systematic methods that included article selection and data extraction conducted by two investigators and summarized findings of multiple outcomes. Additionally, standard tools were used to evaluate the methodological quality of the selected studies (AMSTAR) and the strength of evidence (GRADE). Moreover, most eligible *meta*-analyses were performed based on the results of RCTs, which provided high-quality evidence. However, several limitations of this study should be acknowledged. First, due to the natural shortcomings of RCTs, the sample size of each study was relatively small, we would like to address this problem in the future by seeing results of more RCTs with larger cohort size and update this study. Second, dose‒response analysis could hardly be seen in these studies. The major comparison was sildenafil vs. placebo, and the discrepancy in sildenafil dose varied between studies, which may weaken the consistency of our findings. Finally, we included only published *meta*-analyses in this umbrella review, and studies unpublished or published recently might be omitted.

## 5 Conclusion

After comprehensive review of all existing pooled evidence, we concluded that sildenafil played an important role in systems other than urogenital system, especially its vascular protection effect. This finding provides a sound foundation for further expansion of sildenafil utilization in other diseases. In addition, further studies on more potent mechanisms of sildenafil are guaranteed to support this expansion of sildenafil utilization.

## Data Availability

The original contributions presented in the study are included in the article/Supplementary Material, further inquiries can be directed to the corresponding authors.
