# Supplementary material for: Function of sildenafil on diseases other than urogenital system: An umbrella review
Source: Front Pharmacol. 2023 Feb 6;14:1033492. doi: 10.3389/fphar.2023.1033492 (PMC9939646; doi:10.3389/fphar.2023.1033492)
Supplement: Supplementary file 1 [file Table2.DOCX]

Supplementary Table 1. Nonsignificant effect of sildenafil on multiple diseases.

| Outcome | DOI of Studies | Population | No. of experimental/control | MA metrics | Estimates | 95%CI | No. of studies | RCT | Observational | Effects model | I^2^; Q test P value | Egger test P value |
| --- | --- | --- | --- | --- | --- | --- | --- | --- | --- | --- | --- | --- |
| 6-minute walk distance (meters) | 10.1136/thoraxjnl-2012-202040 | fibrotic interstitial lung disease | 180/198 | MD | 5.25 | -8.90, 19.4 | 2 | 2 |  | Random | 56.6%;0.129 |  |
| Borg score at rest | 10.1136/thoraxjnl-2012-202040 | fibrotic interstitial lung disease | 206/161 | MD | -0.34 | -0.82, 0.13 | 3 | 2 | 1 | Random | 39.5%; 0.198 |  |
| Survey scale (SF-36) | 10.1016/j.lfs.2020.118001 | COPD with PH | 47/41 | MD | 2.64 | -6.85, 12.14 | 2 | 2 |  | Random | 0.11; 60% |  |
| Borg-dyspnea index | 10.1016/j.lfs.2020.118001 | COPD with PH | 95/97 | MD | -0.28 | -1.08, 0.52 | 3 | 3 |  | Random | 0.13; 52% |  |
| Mean pulmonary arterial blood  Pressure (end of therapy) (mmHg) | 10.1002/ppul.25444 | neonates | 46/41 | SMD | 0.13 | -0.30, 0.56 | 2 | 2 |  | Random | 76.2; 0.40 |  |
| Alveolar‐arterial oxygen  difference 24h (mmHg) | 10.1002/ppul.25444 | neonates | 32/25 | SD | -0.17 | -0.70, 0.36 | 2 | 2 |  | Fixed | 67.4%; 0.08 |  |
| Pulmonary capillary wedge pressure (mmHg) | 10.1038/hr.2015.73 | PH due to left heart disease | 32/32 | WMD | -1.0 | -2.30, 0.3 | 2 | 2 |  | Fixed | 0%, 1.0 | 0.13 |
| Alveolar‐arterial oxygen  difference 24h (mmHg) | 10.1002/14651858.CD005494.pub4 | Infants with PH | 32/25 | MD | 1.59 | -18.98, 22.16 | 2 | 2 |  | Random | 74%; 0.05 | 0.88 |
| pre- and intra-operative mean pulmonary arterial pressure (mmHg) | 10.14740/cr962 | PH undergoing cardiac surgery | 46/57 | MD | -7.41 | -21.26, 6.44 | 2 | 2 |  | Random | 89%; 0.003 | 0.29 |
| pre- and intra-operative pulmonary vascular resistance (Wood unit m^2^) | 10.14740/cr962 | PH undergoing cardiac surgery | 56/57 | MD | -8.91 | -84.02, 66.21 | 2 | 2 |  | Random | 0%; 0.56 | 0.82 |
| pre- and intra-operative systemic vascular resistance ( dyn·s·cm^-5^) | 10.14740/cr962 | PH undergoing cardiac surgery | 56/57 | MD | -43.95 | -264.68, 176.79 | 2 | 2 |  | Random | 0%; 0.88 | 0.7 |
| pre- and intra-operative mean arterial pressure ( dyn·s·cm^-5^) | 10.14740/cr962 | PH undergoing cardiac surgery | 56/57 | MD | 1.94 | -5.49, 9.37 | 2 | 2 |  | Random | 52%; 0.15 | 0.61 |
| Pulmonary capillary wedge pressure (mmHg) | 10.1002/ejhf.47 | PH secondary to chronic systolic  heart failure | 48/48 | WMD | -2.54 | -5.82, 0.74 | 3 | 3 |  | Random | 69%; 0.04 | 0.13 |
| Systolic blood pressure (mmHg) | 10.1002/ejhf.47 | PH secondary to chronic systolic  heart failure | 46/45 | WMD | 1.47 | -1.11, 4.04 | 2 | 2 |  | Random | 0%; 0.35 | 0.26 |
| Diastolic blood pressure (mmHg) | 10.1002/ejhf.47 | PH secondary to chronic systolic  heart failure | 46/45 | WMD | -0.31 | -4.06, 3.43 | 2 | 2 |  | Random | 58%; 0.12 | 0.87 |
| Heart rate (p/min) | 10.1002/ejhf.47 | PH secondary to chronic systolic  heart failure | 71/71 | WMD | 1.50 | -2.07, 5.07 | 4 | 4 |  | Random | 80%; 0.002 | 0.41 |
| Systemic vascular resistance ( dyn·s·cm^-5^) | 10.1002/ejhf.47 | PH secondary to chronic systolic  heart failure | 48/48 | WMD | 8.74 | -95.94, 113.42 | 3 | 3 |  | Random | 0%; 0.97 | 0.87 |
| Mechanical ventilation time (days) | 10.1017/S104795112000311X | paediatric | 237/264 | SMD | -0.43 | -0.69, 0.16 | 11 | 11 |  | Fixed |  |  |
| length of ICU (days) | 10.1017/S104795112000311X | paediatric | 190/203 | SMD | -0.43 | -0.7, 0.17 | 9 | 9 |  | Fixed |  |  |
| incidence of pulmonary hypertensive crisis | 10.1017/S104795112000311X | paediatric | 80/96 | RR | 1.51 | 0.124, 18.43 | 4 | 4 |  | Fixed |  |  |
| length of hospital stay (days) | 10.1017/S104795112000311X | paediatric | 78/82 | SMD | -0.27 | -0.6, 0.06 | 3 | 3 |  | Fixed |  |  |
| mortality before discharge | 10.1536/ihj.17-548 | children with PH secondary to congenital heart disease | 122/116 | RR | 0.36 | 0.09, 1.38 | 5 | 5 |  | Fixed | 24%; 0.25 | 0.14 |
| time on the length of hospitalization (days) | 10.1536/ihj.17-548 | children with PH secondary to congenital heart disease | 87/88 | MD | -0.06 | -2.01, 1.88 | 3 | 3 |  | Fixed | 62%; 0.07 | 0.95 |
| heart rate (p/min) | 10.1016/j.ijcard.2014.01.102 | HF | 90/89 | SMD | 0.61 | -2.70, 3.91 | 4 | 4 |  | Random | 71%; 0.01 | 0.72 |
| systolic blood pressure (mmHg) | 10.1016/j.ijcard.2014.01.102 | HF | 155/153 | SMD | -0.75 | -3.78, 2.27 | 5 | 5 |  | Fixed | 0%; 0.91 | 0.63 |
| diastolic blood pressure (mmHg) | 10.1016/j.ijcard.2014.01.102 | HF | 155/153 | SMD | -0.46 | -2.39, 1.48 | 5 | 5 |  | Fixed | 0%; 0.41 | 0.65 |
| emotional function | 10.1016/j.ijcard.2014.01.102 | HF | 84/83 | SMD | -0.43 | -9.90, 9.04 | 4 | 4 |  | Random | 91%; <0.00001 | 0.93 |
| fatigue | 10.1016/j.ijcard.2014.01.102 | HF | 84/83 | SMD | -3.51 | -2.77, 9.8 | 4 | 4 |  | Random | 86%; <0.0001 | 0.27 |
| Breathless | 10.1016/j.ijcard.2014.01.102 | HF | 84/83 | SMD | -0.7 | -10.60, 9.19 | 4 | 4 |  | Random | 95%; <0.00001 | 0.89 |
| Gestational age at birth (months) | 10.1371/journal.pone.0219732 | pregnant women | 37/44 | WMD | -0.12 | -2.84, 2.59 | 2 | 2 |  | Random | 93%; 0.0002 | 0.93 |
| Umbilical artery pulsatility index (%) | 10.1371/journal.pone.0219732 | pregnant women | 125/153 | WMD | 0.04 | -0.18, 0.27 | 4 | 4 |  | Random | 91%; <0.00001 | 0.7 |
| Indication of delivery due to fetal distress | 10.1371/journal.pone.0219732 | pregnant women | 69/70 | RD | -0.03 | -0.17, 0.12 | 2 | 2 |  | Random | 28%; 0.24 | 0.74 |
| Indication of labor due to maternal laboratory test abnormality | 10.1371/journal.pone.0219732 | pregnant women | 69/70 | RD | 0.00 | -0.14, 0.15 | 2 | 2 |  | Random | 0%; 0.32 | 0.97 |
| Indication of delivery due to imminent eclampsia | 10.1371/journal.pone.0219732 | pregnant women | 69/70 | RD | -0.05 | -0.20, 0.10 | 2 | 2 |  | Random | 0%; 0.89 | 0.49 |
| Neonatal mortality | 10.1371/journal.pone.0219732 | pregnant women | 294/304 | RD | -0.01 | -0.05, 0.03 | 7 | 7 |  | Random | 0%; 0.88 | 0.67 |
| middle cerebral artery pulsatility index (%) | 10.1016/j.ejogrb.2021.10.032 | pregnancies with fetal growth restriction | 260/267 | WMD | 0.30 | -0.64, 1.25 | 5 | 5 |  | Random | 99.8; <0.001 |  |
| Change of HbA1c | 10.1016/j.jcte.2016.11.003 | type 2 diabetes mellitus | 100/98 | SMD | 0.17 | -0.64, 0.97 | 4 | 4 |  | Random | 53%; 0.1 | 0.68 |
| endothelin 1 serum levels (ng/L) | 10.1530/EJE-14-0700 | type 2 diabetes mellitus | 40/39 | WMD | -0.94 | -5.49, 3.62 | 2 | 2 |  | Random | 1%; 0.32 | 0.69 |
| high sensitivity C-reactive protein plasma level (mg/l) | 10.1530/EJE-14-0700 | type 2 diabetes mellitus | 30/30 | WMD | -0.36 | -0.91, 0.19 | 2 | 2 |  | Random | 0%; 0.64 | 0.2 |
| interleukin 6 serum level (pg/ml) | 10.1530/EJE-14-0700 | type 2 diabetes mellitus | 163/162 | WMD | -0.82 | -1.58, 0.07 | 2 | 2 |  | Random | 96%; <0.00001 | 0.03 |
| Colorectal cancer risk | 10.1007/s00384-021-04022-5 |  | 197540/257948 | RR | 0.85 | 0.57, 1.27 | 2 |  | 2 | Random | 71.8%; 0.06 | 0.428 |

Abbreviations: COPD Chronic obstructive pulmonary disease, PH pulmonary arterial hypertension, HF heart failure, WMD Weighted mean difference, SMD Standard mean difference, RR risk ratio, RD Risk difference, SD Standard Difference, MD Mean difference.

Supplementary Table 2. Assessments of AMSTAR scores for studies included.

| Ref. No. | Author | Year | A priori design provided | Duplicate study selection & data extraction | At least two electronic databases searched | Status of  publication used as an inclusion criterion | List of  included and excluded studies provided | Characteristics of included  studies provided | Scientific quality of  included studies assessed | Scientific quality of the included studies used  appropriately to form  conclusions | Appropriate methods to  combine studies | Publication bias assessed | Conflict of interest included | Total AMSTAR Score |
| --- | --- | --- | --- | --- | --- | --- | --- | --- | --- | --- | --- | --- | --- | --- |
| 13 | Hao Y et al. | 2018 | 0 | 1 | 1 | 0 | 1 | 1 | 0 | 1 | 1 | 1 | 1 | 8 |
| 24 | He B et al. | 2010 | 0 | 1 | 1 | 1 | 0 | 1 | 1 | 0 | 1 | 1 | 0 | 7 |
| 17 | He Z et al. | 2021 | 0 | 1 | 1 | 0 | 1 | 1 | 0 | 1 | 1 | 1 | 1 | 8 |
| 15 | Jiang R et al. | 2015 | 0 | 1 | 1 | 0 | 1 | 1 | 0 | 1 | 1 | 1 | 1 | 8 |
| 16 | Kanthapillai P et al. | 2019 | 0 | 1 | 1 | 0 | 1 | 1 | 0 | 0 | 1 | 0 | 1 | 6 |
| 22 | Kelly LE et al. | 2020 | 0 | 1 | 1 | 0 | 1 | 1 | 1 | 0 | 1 | 1 | 1 | 8 |
| 21 | Villanueva DLE et al. | 2019 | 0 | 1 | 1 | 0 | 1 | 1 | 0 | 1 | 1 | 0 | 1 | 7 |
| 19 | Wu X et al. | 2014 | 0 | 1 | 1 | 0 | 1 | 1 | 0 | 1 | 1 | 1 | 1 | 8 |
| 18 | Zhang Q et al. | 2020 | 0 | 1 | 1 | 0 | 1 | 1 | 0 | 0 | 1 | 1 | 1 | 7 |
| 25 | Jiang L et al. | 2018 | 0 | 0 | 1 | 0 | 1 | 1 | 0 | 0 | 1 | 0 | 1 | 5 |
| 14 | Zhuang XD et al. | 2014 | 0 | 1 | 1 | 0 | 1 | 1 | 0 | 0 | 1 | 0 | 0 | 5 |
| 20 | Carter EA et al. | 2019 | 0 | 0 | 1 | 0 | 1 | 1 | 0 | 0 | 1 | 1 | 1 | 6 |
| 26 | Ferreira R et al. | 2019 | 1 | 1 | 1 | 0 | 1 | 1 | 1 | 1 | 1 | 1 | 1 | 10 |
| 27 | Hessami K et al. | 2021 | 1 | 1 | 1 | 0 | 1 | 1 | 0 | 0 | 1 | 1 | 1 | 8 |
| 28 | Santi D et al. | 2014 | 0 | 0 | 1 | 0 | 1 | 1 | 1 | 0 | 1 | 0 | 1 | 6 |
| 23 | Chen X et al. | 2015 | 0 | 1 | 1 | 1 | 1 | 1 | 0 | 1 | 1 | 1 | 1 | 9 |
| 29 | Han X et al. | 2018 | 0 | 1 | 1 | 0 | 1 | 1 | 0 | 1 | 1 | 1 | 1 | 8 |

Supplementary Table 3. GRADE classification of quality of evidence for Sildenafil therapeutic effects.

| Outcome | Certainty assessment | | | | | | | Certainty |
| --- | --- | --- | --- | --- | --- | --- | --- | --- |
|  | No. of studies | Ref. No. | Risk of bias | inconsistency | indirectness | imprecision | Publication bias |  |
| Exercise capacity | 4 | 23 | Serious | Serious | Not serious | Not serious | No | ⨁⨁◯◯ Low |
| 6MWD | 9 | 13 | Not serious | Serious | Not serious | Serious | Strongly suspected | ⨁◯◯◯ Very low |
| PASP | 7 | 13 | Not serious | Serious | Not serious | Not serious | Strongly suspected | ⨁⨁◯◯ Low |
| clinical worsening | 3 | 24 | Not serious | Not serious | Not serious | Not serious | Strongly suspected | ⨁⨁⨁◯ Moderate |
| mPAP | 2 | 15 | Not serious | Not serious | Not serious | Not serious | Strongly suspected | ⨁⨁⨁◯ Moderate |
| PVR | 2 | 15 | Not serious | Not serious | Not serious | Serious | Strongly suspected | ⨁⨁◯◯ Low |
| PASP-6m | 4 | 15 | Not serious | Serious | Not serious | Not serious | Strongly suspected | ⨁⨁◯◯ Low |
| PASP | 2 | 16 | Not serious | Serious | Not serious | Serious | Strongly suspected | ⨁◯◯◯ Very low |
| Oxygenation index for neonates | 2 | 22 | Not serious | Not serious | Not serious | Not serious | No | ⨁⨁⨁⨁ High |
| Mean airway pressure (24h) | 2 | 22 | Not serious | Not serious | Not serious | Not serious | No | ⨁⨁⨁⨁ High |
| pre- and intra-operative sPAP | 3 | 21 | Not serious | Serious | Not serious | Serious | No | ⨁⨁◯◯ Low |
| pre- and post-operative sPAP | 2 | 21 | Not serious | Not serious | Not serious | Not serious | No | ⨁⨁⨁⨁ High |
| Mean PAP | 3 | 19 | Not serious | Very serious | Not serious | Serious | Strongly suspected | ⨁◯◯◯ Very low |
| Hospitalization | 4 | 19 | Not serious | Not serious | Not serious | Serious | Strongly suspected | ⨁⨁◯◯ Low |
| LVEF | 3 | 19 | Not serious | Serious | Not serious | Not serious | Strongly suspected | ⨁⨁◯◯ Low |
| VE/VO_2_ slope | 5 | 19 | Not serious | Serious | Not serious | Not serious | Strongly suspected | ⨁⨁◯◯ Low |
| Breathless | 3 | 19 | Not serious | Not serious | Not serious | Not serious | Strongly suspected | ⨁⨁⨁◯ Moderate |
| Fatigue | 3 | 19 | Not serious | Not serious | Not serious | Serious | Strongly suspected | ⨁⨁◯◯ Low |
| Emotional function | 3 | 19 | Not serious | Not serious | Not serious | Not serious | Strongly suspected | ⨁⨁⨁◯ Moderate |
| Mortality | 11 | 18 | Not serious | Not serious | Not serious | Not serious | No | ⨁⨁⨁⨁ High |
| Mean PAP | 8 | 随机试验 | Not serious | Serious | Not serious | Serious | No | ⨁⨁◯◯ Low |
| Length of ICU stay | 3 | 25 | Not serious | Not serious | Not serious | Very serious | No | ⨁⨁◯◯ Low |
| peak VO_2_ | 6 | 14 | Not serious | Serious | Not serious | Not serious | No | ⨁⨁⨁◯ Moderate |
| VO_2_ at AT | 3 | 14 | Not serious | Not serious | Not serious | Not serious | Strongly suspected | ⨁⨁⨁◯ Moderate |
| VE/CO_2_ slope | 4 | 14 | Not serious | Not serious | Not serious | Not serious | Strongly suspected | ⨁⨁⨁◯ Moderate |
| LV ejection fraction in HFrEF | 2 | 14 | Not serious | Not serious | Not serious | Not serious | Strongly suspected | ⨁⨁⨁◯ Moderate |
| LV ejection fraction in HF | 3 | 14 | Not serious | Not serious | Not serious | Not serious | Strongly suspected | ⨁⨁⨁◯ Moderate |
| PASP in HFrEF | 5 | 14 | Not serious | Serious | Not serious | Not serious | Strongly suspected | ⨁⨁◯◯ Low |
| PASP in HF | 5 | 14 | Not serious | Serious | Not serious | Not serious | Strongly suspected | ⨁⨁◯◯ Low |
| PAP during rest | 7 | 20 | Not serious | Serious | Not serious | Not serious | Strongly suspected | ⨁⨁◯◯ Low |
| PAP during exercise | 4 | 20 | Not serious | Serious | Not serious | Not serious | Strongly suspected | ⨁⨁◯◯ Low |
| Cardiac output during exercise | 9 | 20 | Not serious | Not serious | Not serious | Not serious | Strongly suspected | ⨁⨁⨁◯ Moderate |
| Cardiac output during rest | 4 | 20 | Not serious | Serious | Not serious | Not serious | Strongly suspected | ⨁⨁◯◯ Low |
| SPO_2_ | 15 | 20 | Not serious | Serious | Not serious | Not serious | Strongly suspected | ⨁⨁◯◯ Low |
| Performance | 8 | 20 | Not serious | Serious | Not serious | Not serious | Strongly suspected | ⨁⨁◯◯ Low |
| Fetal weight | 5 | 26 | Not serious | Serious | Not serious | Not serious | Strongly suspected | ⨁⨁◯◯ Low |
| UtA-PI | 3 | 27 | Not serious | Not serious | Not serious | Not serious | No | ⨁⨁⨁⨁ High |
| UA-PI | 6 | 27 | Not serious | Serious | Not serious | Not serious | No | ⨁⨁⨁◯ Moderate |
| FMD values | 4 | 28 | Not serious | Serious | Not serious | Not serious | No | ⨁⨁⨁◯ Moderate |
| Melanoma risk | 6 | 29 | Not serious | Serious | Not serious | Not serious | Strongly suspected | ⨁⨁◯◯ Low |
